# A picture of trends in Aujeszky’s disease virus exposure in wild boar in the Swiss and European contexts

**DOI:** 10.1186/s12917-015-0592-5

**Published:** 2015-11-07

**Authors:** Roman Kaspar Meier, Francisco Ruiz-Fons, Marie-Pierre Ryser-Degiorgis

**Affiliations:** Centre for Fish and Wildlife Health (FIWI), Vetsuisse Faculty, University of Bern, Bern, Switzerland; SaBio group, Instituto de Investigación en Recursos Cinegéticos IREC (CSIC-UCLM-JCCM), Ciudad Real, Spain

**Keywords:** Europe, Herpesvirus, Pseudorabies, *Sus scrofa*, Serosurvey, ELISA, Review, Switzerland

## Abstract

**Background:**

In parallel to the increase of wild boar abundance in the past decades, an increase of exposure to the Aujeszky’s disease virus (ADV) has been reported in wild boar in several parts of Europe. Since high animal densities have been proposed to be one of the major factors influencing ADV seroprevalence in wild boar populations and wild boar abundance has increased in Switzerland, too, a re-evaluation of the ADV status was required in wild boar in Switzerland. We tested wild boar sera collected from 2008–2013 with a commercial ELISA for antibodies against ADV. To set our data in the European context, we reviewed scientific publications on ADV serosurveys in Europe for two time periods (1995–2007 and 2008–2014).

**Results:**

Seven out of 1,228 wild boar sera were positive for antibodies against ADV, resulting in an estimated seroprevalence of 0.57 % (95 % confidence interval CI: 0.32–0.96 %). This is significantly lower than the prevalence of a previous survey in 2004–2005. The literature review revealed that high to very high ADV seroprevalences are reported from Mediterranean and Central-eastern countries. By contrast, an “island” of low to medium seroprevalences is observed in the centre of Europe with few isolated foci of high seroprevalences. We were unable to identify a general temporal trend of ADV seroprevalence at European scale.

**Conclusions:**

The seroprevalence of ADV in wild boar in Switzerland belongs among the lowest documented in Europe. Considering the disparity of seroprevalences in wild boar in Europe, the fact that seroprevalences in Switzerland and other countries have decreased despite increasing wild boar densities and the knowledge that stress leads to the reactivation of latent ADV with subsequent excretion and transmission, we hypothesize that not only animal density but a range of factors leading to stress - such as management - might play a crucial role in the dynamics of ADV infections.

## Background

Aujeszky’s disease (AD) or Pseudorabies is an economically important disease of domestic swine that causes substantial losses to the pig industry worldwide, due to decrease of productivity and trade restrictions [1]. In several European countries and North America AD does not occur in domestic swine owing to successful eradication programs [2, 3].

AD is caused by Aujeszky’s disease virus (ADV) (syn. Suid Herpesvirus 1 or Pseudorabies virus), a Varicellovirus of the Herpesviridae family, subfamily Alphaherpesvirinae [4]. The only natural hosts of the virus are Suidae (*Sus scrofa scrofa*) including domestic swine, wild boar and their hybrids. In domestic swine the virus leads to varying clinical courses including high mortality and disorders of the respiratory, reproductive and central nervous systems [5]. Most other mammals (ungulates, carnivores, lagomorphs and rodents) are susceptible to infection but they represent dead-end hosts and die from infection [6]. Higher primates including humans are not susceptible to ADV [7]. A negative impact of ADV infections on free-ranging wild boar populations has not yet been demonstrated, except for two reported AD outbreaks [8, 9]. Experimental infections of wild boar with ADV showed that clinical signs depend on the virulence of the strain and the viral dose [10]. Characterized isolates of ADV from wild boar mostly belong to the genotype I and are of low virulence, whereas those from domestic swine mostly belong to the genotype II [11]. In agreement with these observations, a study conducted in Spain suggested that ADV seroprevalences in domestic pigs are not directly linked to ADV seroprevalences of wild boar in the same region [12]. However, it is widely recognized that free-ranging wild boar can act as an ADV reservoir [1, 12, 13] and it is of concern that transmission from wild boar to domestic swine could occur. Pathogen transmission from wild boar to domestic swine has been documented [14–16] and wild boar have been suspected to be the source of infection for an AD outbreak in domestic pigs in France [13]. In the past decades an increase of ADV seroprevalences has been observed in European wild boar [1, 3], locally reaching very high levels (e.g. 100 % in Spain) [17]. The dramatic increase of wild boar abundance in Europe during the same period [18] may have contributed to this process because high ADV seroprevalences seem to be associated with high wild boar population densities [19] and wild boar aggregation [20].

In parallel to the increasing ADV seroprevalences in wild boar, an increase of hunting dogs dying of AD after contact with hunted wild boar has occurred [21–26]. Furthermore, reports of fatal spillover of ADV on captive wild felids and canids after feeding on infected wild boar carcasses suggests that increased ADV occurrence in wild boar may represent a potential threat for protected large carnivores [27–29]. Therefore surveillance of ADV in wild ranging wild boar is strongly recommended [1, 3, 19, 30].

In Switzerland, a serosurvey of ADV in free-ranging wild boar performed in 2004/2005 revealed a seroprevalence of only 2.8 % (95 % confidence interval (CI): 1.9-4.0 %) [31]. Since then, hunting bag data have further indicated an increase in wild boar abundance and possibly densities [30] like elsewhere in Europe. Therefore, it has become of concern that ADV infection prevalence may have also increased.

The aims of this study were (i) to re-evaluate the status of ADV in the Swiss wild boar population using the methods recommended by the EMIDA-Eranet project APHAEA [32] and (ii) to compare our data with those from other European wild boar populations, considering two time periods (1995–2007 and 2008–2014).

## Results

### Serosurvey in Switzerland

**Fig. 1:**
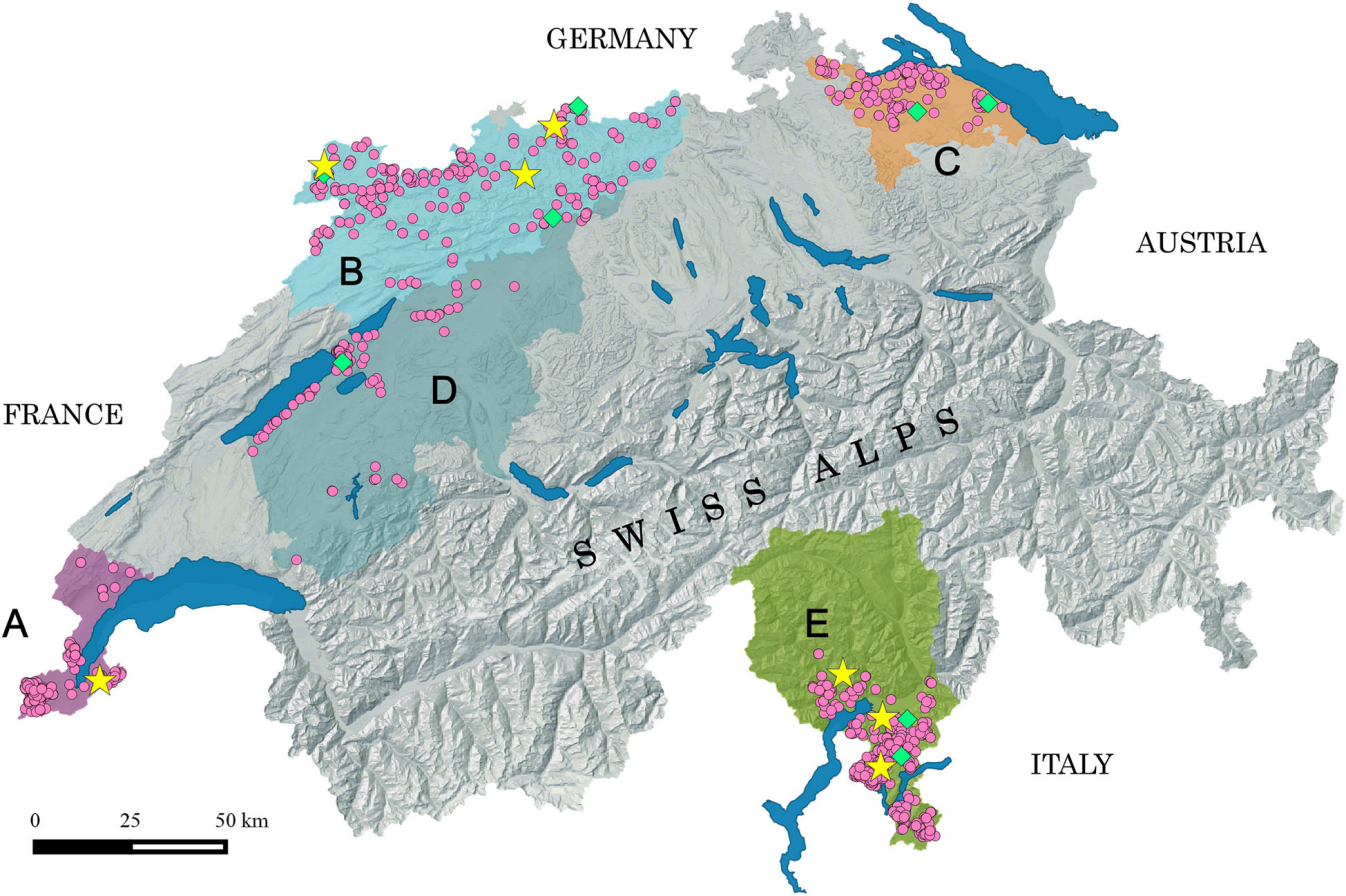
Map of Switzerland showing the study units and the origin of sampled wild boar. Shades of grey refer to the landscape relief and main lakes are indicated in plain blue. Letters and transparent colored surfaces refer to the five study units: *Purple*, **a** = Geneva; *Light blue*, **b** = Jura Mountains; *Orange*, **c** = Thurgovia; *Grey-blue*, **d** = Swiss plateau; *Green*, **e** = Ticino. Sera were tested by ELISA for antibodies against ADV. Colored dots, stars and diamonds indicate the location of the sampled wild boar (2008-2013): Pink dots = seronegative; Yellow stars = seropositive; Green diamonds = doubtful

Seven of 1,228 wild boar blood samples tested by enzyme linked Immunosorbent assay (ELISA) had antibodies against ADV, and the result of eight serum samples remained doubtful despite repeated testing. We obtained an overall estimated antibody prevalence of 0.57 % (95 % confidence interval CI: 0.32-0.96 %). This represents a significant decrease of seroprevalence (*P* = <0.001) in the Swiss wild boar population since the last serosurvey in 2004/2005 (2.83 %, 95 % CI: 1.91-4.02 %) [31]. The seven positive animals were of both sexes, of all age classes, from three different study units (A, B, E) and four years (2009, 2011, 2012, 2013) (Fig. 1). There were no significant differences among these categories or between the two wild boar populations (north: 0.44 %, 95 % CI: 0.2-0.89 %; south: 0.90 %, 95 % CI: 0.33-2.00 %).

### Review on AD in wild boar in Europe

**Fig. 2:**
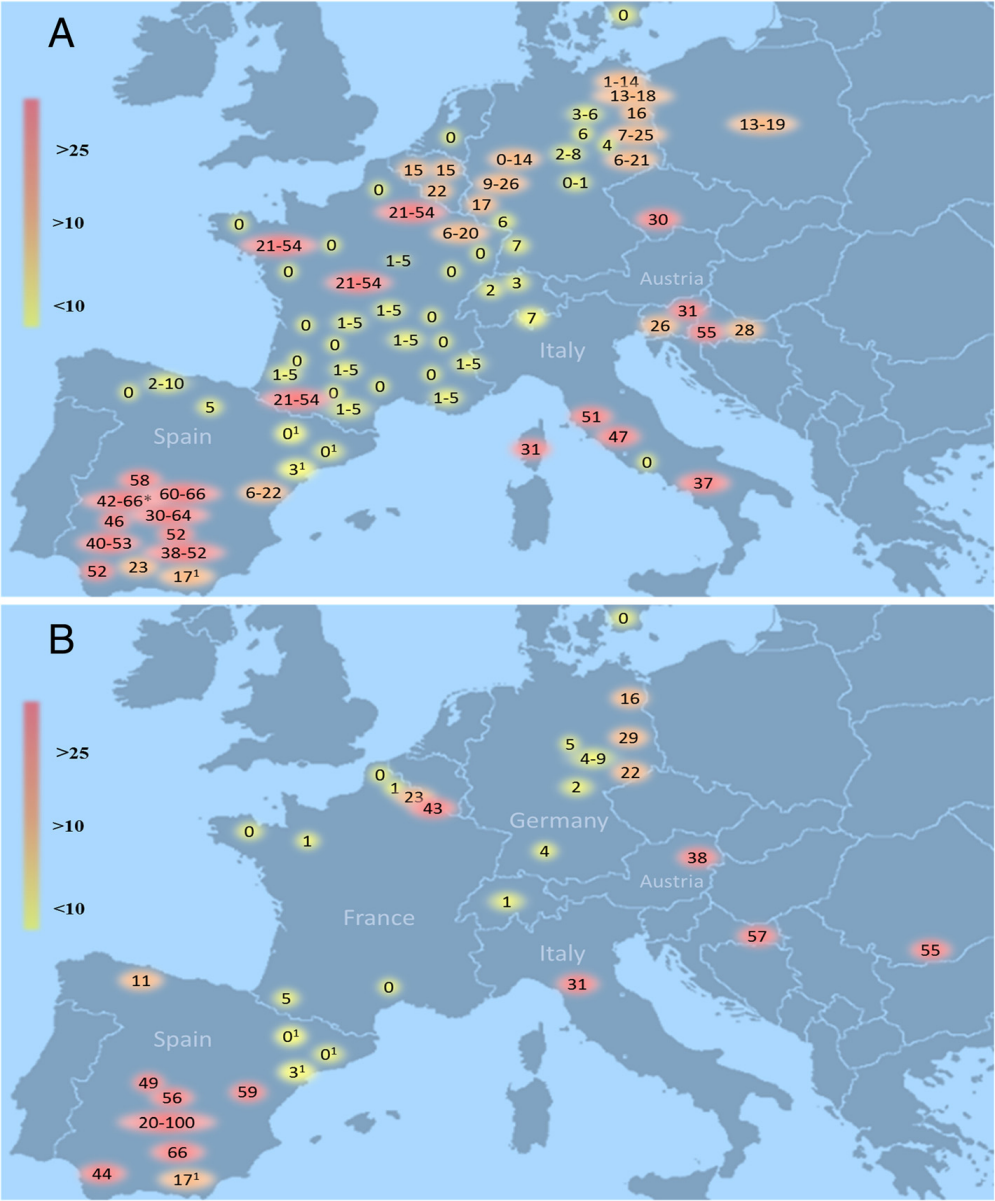
Seroprevalence of ADV in free-living wild boar in Europe from 1995-2014. Compilation of published data obtained by ELISA for two time periods: (**a**) 1995-2007 [1, 3, 8, 17, 19, 31, 34, 35, 37–43, 45–50, 59, 60, 62, 64, 65]; (**b**) 2008-2014 [1, 17, 19, 38, 43, 44, 47, 61, 63, 64, 82]. Numbers refer to estimated seroprevalences for the regions where they are placed.*Fenced animals included. ^1^Data obtained over both time periods

ADV seroprevalences in wild boar populations strongly vary among European regions, ranging from 0 to 100 % [17, 33] (Fig. 2). The highest seroprevalences have been documented in Mediterranean countries including Spain (up to 100 %) [1, 17, 34–39], Italy (up to 51 %) [40, 41] and Croatia (up to 57 %) [42, 43], as well as in Romania (55 %) [44]; followed by central and eastern European countries such as Slovenia (31 %) [45, 46], Austria (38 %) [47], Czech Republic (30 %) [48] and northeastern Germany (up to 29 %) [19, 49]. In contrast, there is an area with low to moderate ADV seroprevalences in the centre and north of Europe: Switzerland (<4 %) [31, 50], the Netherlands (0 %) [51–53], Sweden (0 %) [33, 54–58], parts of France [13, 59–61] and of Germany [19, 49, 62–64]. Within this area of low seroprevalences, multiple regions with higher seroprevalences exist: Although the overall seroprevalence of continental France lies at 6 %, several provinces in the centre (Le Loir-et-Cher, le Loiret), in the northwest (l’Ille-et-Villaine), in the Mediterranean area (Corse) and the north-east of France (les Ardennes, la Meuthe-et-Moselle, la Meuse) reach levels between 21 and 54 % [60]. The provinces in the northeast of France seem to belong to a transnational wild boar population with moderate to high seroprevalences in Luxembourg (17 %) [65], Belgium (15-22 %) [65] and western Germany (9-26 %) [62]. Similar situations of strongly heterogeneous seroprevalences within the same country exist also in Spain [1, 37–39] and Italy [3].

While there is a good to very good data coverage of western Europe during the first time period, there is a lack of information for large parts of Europe during the second time period. Moreover, recent data partially originate from different geographical areas than those collected during the first period (Fig. 2), making comparisons difficult. Where such comparisons are possible, all conceivable courses are observed: decreasing in southwestern France [60, 61], stable-high in Spain [1] and increasing in Germany and Croatia [19, 43, 64]. A general pan-European trend was not detected due to this varying regional evolution of the seroprevalences.

## Discussion

The regional increase of ADV seroprevalence in various wild boar populations in Europe and the increasing number of reports of hunting dogs dying of ADV after exposure to ADV infected wild boar required a re-evaluation of the ADV status of wild boar populations in Switzerland. This study provides current seroprevalence data for Switzerland and sets the obtained results in a European context, examining published data from two time periods.

In Switzerland, domestic pigs have been officially free of AD since 2001 and there has been no report of AD in other species either during the past decade [66]. The obtained overall seroprevalence in wild boar was very low, which suggests that ADV infections only sporadically occur in wild boar populations in Switzerland. Compared to the results of the last serosurvey in 2004/2005, we documented a significant decrease from 2.8 % to 0.6 %. This decrease would also be observed if doubtful results were classified as positive (estimated prevalence of 1.2 %). Furthermore, the difference between the previous and present study is enhanced by the fact that seroprevalence had previously been estimated after applying a virus neutralization test on the ELISA-positive samples [31], thus increasing specificity but reducing sensitivity compared to our present results.

The seroprevalence estimated for Switzerland remains one of the lowest in Europe. The literature review revealed an inhomogeneous situation at continental scale and over time, with an “island” of low seroprevalences in central Europe, surrounded by medium to high seroprevalences in southern and central-eastern regions. This rough pattern together with the general inhomogeneity of seroprevalences at smaller scale raises the question of the major factors influencing ADV transmission among wild boar.

Wild boar density has been proposed as a factor influencing ADV seroprevalence [1, 19, 37, 39]. A comprehensive long-term study in eastern Germany showed a correlation between ADV seroprevalence and the “hunting index of population density” (HIPD, i.e. number of wild boar shot/km^2^/year) [19]. In south-central Spain, where ADV seroprevalence in wild boar is particularly high, the wild boar is intensively managed for hunting purposes [1, 20]. Fencing, artificial feeding and translocations [35, 37] lead to extremely high animal densities of up to 90 individuals/100 ha and to a marked aggregation of wild boar around feeders [67]. Additionally, the scarcity of water in dry habitats results in animal aggregation around water holes [67]. However, high ADV seroprevalences have also been reported from other areas of Europe, e.g. north-eastern Germany, where industrial wild boar management is apparently uncommon. Furthermore, a general pan-European increase of ADV seroprevalence has not been observed, although a dramatic increase of wild boar has occurred in most parts of Europe since the 1950s, resulting in a wider distribution and higher densities of wild boar populations [3]. For example, high wild boar densities are associated with a low ADV seroprevalence in Catalonia in northern Spain [38]. Furthermore, it was documented in Germany that ADV spread in free-ranging wild boar is characterized by an inhomogeneous pattern with cluster formation [64]. Overall, these observations suggest that additionally to animal densities, other factors influence ADV prevalence in wild boar.

Intensified intraspecies contacts resulting from aggregation due to a range of factors (e. g. related to wildlife management, climate or social interactions) are expected to favor virus transmission. However, pathogen characteristics may also play a crucial role in this process. Since seropositive animals are infected lifelong by ADV [4], virus-carrying animals must exist in Switzerland and other regions with low seroprevalence. This raises the question as whether these animals shed the virus or not. Excretion of ADV and resulting infectiousness normally occur within several weeks after infection. However, Herpesviridae have the ability to undergo a latency in sensory ganglia, which inhibits the permanent replication and excretion of the virus [4, 68]. The virus may be reactivated later but this reactivation requires a modulation of the immune system, e.g. by a stressful experience [69–72]. Indeed, treatment of laboratory mice, domestic pigs and wild boar with immunosuppressive drugs such as dexamethasone, results in reactivation and excretion of ADV [10, 69, 73, 74]. Identified stressors enhancing ADV activity include concomitant disease conditions, transport, poor animal husbandry and farrowing in domestic pigs [69], as well as restraint, exposure to cold, and transport in laboratory mice [75]. In wild boar, mating has been proposed as possible source of stress generating ADV venereal excretion [76].

Considering the epidemiological picture of ADV infection in wild boar in Europe and the properties of ADV as a herpesvirus, we propose that factors causing stress may play a major role in the spread and distribution of ADV in wild boar populations. High animal densities, aggregation, overabundance, lack of possibilities to retreat, competition for food, confinement (e.g. fencing), high environmental temperatures, translocations, co-infections with other pathogens, as well as high hunting pressure, drive hunts, and other kinds of disturbance all represent conceivable sources of stress. However, to date it is not possible to identify associations between ADV seroprevalences and such stress factors across Europe due to the lack of information on population management and the inhomogeneity of data on wild boar abundance.

## Conclusions

ADV seroprevalence in wild boar in Switzerland has remained low since the last study and is among the lowest in Europe. Therefore, we had to reject our hypothesis that ADV seroprevalence would have increased in Switzerland in recent years. Moreover, we documented a general heterogeneity of estimated seroprevalences among countries which suggests that wild boar abundance alone does not explain the patterns of ADV spread. We propose that stress-inducing factors leading to reactivation of the latent virus may play a major role in the spread and maintenance of the virus in the wild. Harmonized methods in wildlife health surveillance and ecology, and risk factor analyses for ADV exposure, infection and shedding patterns in European wild boar populations are required to better understand ADV dynamics at the wildlife-domestic animal interface and design adequate disease control measures.

## Methods

### Study area

We selected five different study units (A-E, Fig. 1) in Switzerland (41,284 km^2^) with the aims of: (1) covering the main wild boar habitat; (2) including northern and southern wild boar populations; (3) covering all representative bioregions of Switzerland, i.e. i) the Jura mountains (approx. 4,307 km^2^), shaped by forests and pastures, ii) the densely populated Swiss Plateau (approx. 11,168 km^2^), iii) the Alps (approx. 23,000 km^2^), of which a large part reaches altitudes above the timber line, and iv) the part of Ticino located south from the Alps (approx. 2,812 km^2^); (4) covering most of the Swiss border to France, Germany and Italy; and (5) complementing former studies on wild boar pathogens in Switzerland [77, 78]. Contacts are possible among wild boar in the study units A-D (i.e., northern population) whereas wild boar in study unit E (Ticino, i.e., southern population) are separated from the northern population by the Alps and can only interact with Italian wild boar populations.

### Sample collection and laboratory analysis

Blood samples collected from 1,228 wild boar over six hunting seasons (2008–2013) were available for this study. In accordance with the national hunting law [54] a hunting season was defined as lasting from July 1^st^ to June 30^th^ of the following year, with most of the hunting bag being harvested from December to February. Samples from wild boar shot before 2012 had been collected in the frame of former projects [30, 79] and stored in the archive of the Centre for Fish and Wildlife Health (FIWI Bern, Switzerland), while samples from 2012–2013 were collected for the purpose of the present study. Calculation of the target sample size per hunting season and study unit was derived from the regional hunting bags and performed with the WinEpiscope 2.0 software package. Since 2011 samples sizes have been calculated with the aim of estimating prevalence and assuming a prevalence of 50 %, with a confidence level of 95 % and an accepted absolute error of 5 % [78]. Efforts were made towards an even age and sex distribution among units. Blood samples were collected either by local hunters and game wardens with provided sampling kits and sent to the FIWI or were obtained by FIWI collaborators at game check points. Blood was collected from the thoracic cavity or the cavernous sinusoid [80].

This study did not involve purposeful killing or capture of animals and was exempt from ethical approval according to Swiss legislation. Samples originated from dead wild boar either shot for population regulation purposes (regular hunt, culling by professional game-wardens; 922.0 hunting law) or killed in traffic accidents. Nine samples originated from wild boar found dead submitted to the FIWI for pathological examination.

Information on weight, sex and body condition of the animals as well as the location, circumstances (found dead, hunted or culled) and date of sampling were systematically collected with a standardized datasheet. According to Hebeisen [81], wild boar were classified into four age classes: Piglets: <20 kg, striped coat, *n* = 64; Juveniles: 20-40 kg, reddish coat, *n* = 342; Subadults: 40-60 kg, black coat, *n* = 370; Adults: >60 kg, black or silver coat, *n* = 385; and no age data were delivered for 67 animals. Sex ratio of the sample was balanced, with 597 males and 611 females. Sex was undetermined for 20 animals.

Blood samples were centrifuged immediately after arrival at the FIWI. Serum aliquots were stored at -20 °C until analysis. Sera were tested for antibodies against ADV with a commercial competitive ELISA kit (IDEXX PRV/ADV gI, IDEXX, Inc., USA) successfully applied in former studies in Spain and Germany [1, 19, 34, 37]. According to the manufacturer’s instructions, samples with a sample/negative (S/N)-value greater than 0.6 and less or equal to 0.7 were classified as doubtful, and samples with S/N-values greater than 0.7 as positive. All doubtful and positive samples were retested with the same ELISA.

### Literature review

We performed a review of internationally available scientific articles about serosurveys of ADV. In a first step, three online databases (PubMed, EBSCOhost and Google Scholar) were searched using the key words “wild boar”, “*Sus scrofa*”, “Aujeszky’s disease” and “pseudorabies”. In a second step, we screened references mentioned in the obtained publications selecting studies conducted between 1995 and 2014 on free-ranging wild boar in Europe and providing seroprevalences obtained by ELISA.

### Data management

Data handling and coding was carried out with Microsoft Office Excel 2010 (Microsoft Corporation, Redmond, Washington, USA). Two time periods were defined, both for the Swiss data and the literature review, starting arbitrarily 20 years ago and using the first year of the wild boar sampling campaign carried out by the FIWI as a threshold: 1995–2007 (historical data) and 2008–2014 (samples available for the current study). Prevalence calculations and statistical tests were performed with the NCSS 2007 software (J. L. Hintze, Kaysville, Utah, USA). Prevalences were calculated assuming test sensitivity and specificity of 100 % and excluding doubtful ELISA results. The Fisher’s exact test (FET) was used to test for differences in seroprevalence among sexes, age classes, hunting seasons, study units and populations (north and south). Level of significance was set at *P* < 0.05.

Maps were designed with the free QGIS- Software (QGIS Development Team, 2012. Versions 1.8.0, 2.0.1 and 2.2.0; QGIS Geographic Information System. Open Source Geospatial Foundation Project, http://qgis.osgeo.org) and Microsoft PowerPoint 2010 (Microsoft Corporation, Redmond, Washington, USA).

## Abbreviations

AD: Aujeszky’s disease
ADV: Aujeszky’s disease virus
ELISA: Enzyme linked Immunosorbent assay
CI: Confidence interval
HIPD: Hunting index of population density
FIWI: Centre for Fish and Wildlife Health
S/N: Sample/negative
FET: Fisher’s exact test
